# Supplementary material for: Interaction of Val66Met BDNF and 5-HTTLPR polymorphisms with prevalence of post-earthquake 27-F PTSD in Chilean population
Source: PeerJ. 2023 Sep 4;11:e15870. doi: 10.7717/peerj.15870 (PMC10484206; doi:10.7717/peerj.15870)
Supplement: Table S1 — (*) Variable created to group the number of forms of maltreatment experienced in childhood, independent of the type of maltreatment (physical, emotional or sexual). (**) Death of a family member, Being trapped under rubble, Having physical injuries that required hospitalisation or were life-threatening, Severe damage to the dwelling or total loss. [file peerj-11-15870-s001.docx]

| **Table S1**  Logistic regression models for factors associated with the development of post-earthquake PTSD | | | | | | |
| --- | --- | --- | --- | --- | --- | --- |
|  |  | Logistic regression model | | | | |
| Variables | Detail | Univariate genetic | More Interaction between polymorphisms | More biological variables | More psychosocial factors model | More earthquake event |
|  |  |  |  |  |  |  |
| Val66Met polymorphism | GG | - | - | - | - | - |
|  | GA-AA | 1.27 (0.84-1.93, p=0.261) | 1.26 (0.77-2.10, p=0.362) | 1.27 (0.77-2.12, p=0.348) | 1.31 (0.79-2.19, p=0.299) | 1.24 (0.73-2.10, p=0.428) |
|  |  |  |  |  |  |  |
| 5HTTLPR polymorphism | Other | - | - | - | - | - |
|  | S’/S’ | 0.81 (0.51-1.26, p=0.355) | 0.79 (0.39-1.53, p=0.505) | 0.80 (0.39-1.55, p=0.517) | 0.80 (0.39-1.56, p=0.526) | 0.82 (0.39-1.62, p=0.574) |
|  |  |  |  |  |  |  |
| Val66Met (GA-AA) - 5HTTLPR (S’/S’) Polymorphic Interaction | Interaction |  | 1.03 (0.42-2.57, p=0.949) | 1.01 (0.41-2.52, p=0.984) | 1.01 (0.41-2.54, p=0.983) | 1.02 (0.82-1.26, p=0.830) |
|  |  |  |  |  |  |  |
|  |  |  |  |  |  |  |
| Sex | Female |  |  | - | - | - |
|  | Male |  |  | 0.75 (0.42-1.27, p=0.313) | 0.80 (0.45-1.37, p=0.444) | 0.72 (0.39-1.27, p=0.273) |
|  |  |  |  |  |  |  |
| Age | Mean (SD) |  |  | 0.99 (0.98-1.01, p=0.345) | 0.99 (0.97-1.00, p=0.104) | 1.00 (0.98-1.01, p=0.586) |
|  |  |  |  |  |  |  |
| Schooling | Illiterate |  |  |  | - | - |
|  | Basic, secondary or higher |  |  |  | 1.24 (0.76-2.04, p=0.381) | 1.38 (0.83-2.31, p=0.209) |
|  |  |  |  |  | 0.62 (0.29-1.23, p=0.191) | 0.68 (0.31-1.36, p=0.297) |
|  |  |  |  |  |  |  |
| Number of forms of maltreatment experienced during childhood* | Mean (SD) |  |  |  | 1.06 (0.85-1.30, p=0.610) | 1.02 (0.82-1.26, p=0.830) |
|  |  |  |  |  |  |  |
| Diagnosis of a concomitant depressive episode | No |  |  |  | - | - |
|  | Yes |  |  |  | **2.32 (1.15-4.37, p=0.013)** | **2.09 (1.02-4.06, p=0.035)** |
|  |  |  |  |  |  |  |
| Experience of a critical traumatic event associated with the earthquake** | No |  |  |  |  | - |
|  | Yes |  |  |  |  | 0.53 (0.26-1.05, p=0.079) |
|  |  |  |  |  |  |  |
| Number of traumatic events experienced / witnessed | Mean (ds) |  |  |  |  | **1.65 (1.41-1.94, p<0.001)** |

(*) Variable created to group the number of forms of maltreatment experienced in childhood, independent of the type of maltreatment (physical, emotional or sexual).

(**) Death of a family member, Being trapped under rubble, Having physical injuries that required hospitalisation or were life-threatening, Severe damage to the dwelling or total loss.
